# Editor's Perspective on Health Economics in Prosthetics and Orthotics

**DOI:** 10.33137/cpoj.v4i2.37135

**Published:** 2021-09-21

**Authors:** S.U. Raschke

**Affiliations:** 1 British Columbia Institute of Technology (BCIT), 3700 Willingdon Avenue, Burnaby, British Columbia, Canada.

**Keywords:** Health Economics, Prosthetics, Orthotics, Rehabilitation Engineering, Business Practices, Health Economic Evaluation, Evidence-Based Practices, Reimbursement

## Abstract

There is a scarcity of literature exploring Health Economics in Prosthetics and Orthotics (P&O). The P&O sector has, over the past decades, moved to a Bachelor's or Masters Degree level as the requirement for entry to practice and, with that, there has been a growing body of research and knowledge generation focusing primarily on clinical aspects and engineering advances. No corresponding body of research has emerged on the economic aspects of P&O, creating a fundamental weakness in both technical and clinical research efforts to advance this field within an economically sustainable framework. This weakness will become critical as data driven engineering advances (e.g. exoskeletons, mass customizable prostheses) and clinical improvements (e.g. osseointegration, diabetes treatments) will make reimbursement for devices ever more complex and challenging. The tension between what is possible and what is fundable will increase unless what is possible also drives down costs. Finding the right balance in Prosthetics and Orthotics will be a challenge, as this sector already struggles to justify current standards of care. This Special Edition takes a snapshot of stakeholder perspectives and opinions on the topic of Health Economics in P&O and is organized around the following stakeholder groups: End-user, Researcher (Engineering and Clinical), Prosthetic and Orthotic Practitioner and, of critical importance, four papers describing an interdisciplinary project on the Health Economics of Osseointegration that was led by a payor. Each author was also asked to provide a “Call to Action” in which they identify one or more key areas that need to be addressed in order to move forward with the barriers or opportunities they have identified in their paper. The intent of the Special Edition is to generate discussion and encourage more in-depth research on this topic.

## BACKGROUND

In preparing this Special Edition, I took time to reflect on the importance of Special Editions in the publishing cycle of academic journals. Special Editions can collect the works emerging from a special event such as a conference or they can fill gaps in the literature where a topic has been underserved but is, nonetheless, worthy of attention. In this case it is the latter that motivated the Editorial Team. The idea for a Special Edition was sparked by a 60+ page long manuscript submitted for comment to the Editors of the Canadian Prosthetics ad Orthotics Journal (CPOJ) by Dr. Laurant Frossard, from the Queensland University of Technology. The submission described a collaborative, interdisciplinary, payor-led project that used a novel, carefully considered methodology that was also applied and practical. The goal of the project was to develop an economic foundation and justification for Osseointegration, which is the primary focus of Dr. Frossard's team. While not suitable for peer-review publication due to its length, density and atypical format, the work this interdisciplinary collaboration did to establish this economic foundation is novel, interesting and directly related to the long-term financial sustainability of the Prosthetics and Orthotics sector. The thinness of literature on this topic sparked a discussion within the Editorial team on the critical need to bring attention to this topic. It was then that we decided the time had come for our first Special Edition.

Over the past 20 years the entry level to practice in Prosthetics and Orthotics shifted to either a Bachelor's or Master's Degree in most parts of the world, and a number of PhD Programs have been established. This shift provided the necessary educational basis to allow prosthetists and orthotists to participate in and lead research and has led to a growth of peer-reviewed knowledge published in academic journals which typically focus on the evaluation of clinical practices, the development of evidence-based measures and the development of more sophisticated, better functioning componentry. However, a knowledge gap persists around objective economic and business-related issues. The provision pathway of Prosthetic and Orthotic care is not typically thought of in economic terms. Nonetheless, the economics of this provision pathway are a strong undercurrent that drives events and decisions both at practical and at policy levels. This knowledge gap creates a fundamental weakness in both technical and clinical research efforts to advance this field. If it is not considered within the research and development process, the long-term economic sustainability of Prosthetics and Orthotics is jeopardized.

Credit must be given to researchers who have already begun to fill this knowledge gap, some of whom have contributed to this Special Edition, but more work needs to be done. The most comprehensive treatment of the topic is the recently published comprehensive systemic literature review of health economic evaluations in Prosthetics and Orthotics done by Clark, Dillon and Shiell, who noted that, to the best of their knowledge, they are the first to have done such a review and that while some published papers exist, they are limited by relatively narrow focus. They point to a need for improving the evidence-based methodologies being used in Prosthetics and Orthotics to meet contemporary standards for rigorous Health Economic Evaluation (HEE) studies.^1–3^

### Need for a discussion on health economics in prosthetics and orthotics

The identification of need for a deeper understanding of the underlying business and economic basis of the practice of Prosthetics and Orthotics comes at a critical time. The accelerating, data driven digital revolution is creating extraordinary opportunities for innovation in medical and rehabilitation technology development, as well as supporting clinical improvements in surgical techniques such as osseointegration, limb reattachment and more effective treatments for diabetes and neuromuscular disorders. Alongside this, innovative software architecture and infrastructure to support new business models are being created, such that services and products can now travel from maker to the customer directly or can be created and delivered as a hybrid virtual model. This evolving ecosystem, driven by objective data, supports the creation of novel prosthetic and orthotic solutions that are likely to become increasingly personalized and diverse and that better meet the end-user's needs. All of these will impact reimbursement models and, in the wake of this disruption of the status quo, payors will likely continue to focus on generating cost savings. The tension between what is possible and what is fundable will increase unless what is possible also drives down costs. Finding the right balance in Prosthetics and Orthotics will be a challenge, as this sector already struggles to justify current standards of care.

This brings us back to the health economics work done by Dr. Frossard and his collaborators, around which this issue is structured. The medical and engineering challenges that have been addressed by Osseointegration researchers worldwide are significant and inspiring. This research challenges society to completely re-think amputation, rehabilitation and life with a prosthesis. But, in attempting to allow amputees to access the benefits brought by this new paradigm, a substantial barrier has to be overcome, namely making a convincing case to payors for the value the approach provides vs. the cost. In the same way, building a sustainable future in Prosthetics and Orthotics, both clinically and technologically, requires a realistic understanding of the economics and business constraints in the sector. Both researchers and practitioners must incorporate economic realities and constraints into their work if they are to have any sort of prospect of that work making a meaningful impact.

Frossard et al were not the first to identify this barrier to novel approaches or technology in Prosthetics and Orthotics. However, they are the first to do a project which included representation from the payor side and to develop an objective methodology that considered carefully a wide range of inputs that can be used to calculate ‘value’ and not just ‘cost’ of the approach. The result is one of the most comprehensive treatments of the topic at this time. Furthermore, this was a project that was led by the payor, which is a critically important differentiator, as payors have typically been silent when it comes to providing transparent, objective criteria which makes clear how they determine what they believe has value/what they will reimburse.

### Response

It was the recognition of the importance of generating a broad starting point for discussion on this topic that led to this Edition on Health Economics in Prosthetics and Orthotics. Because of the absence of an extensive, formal body of research and researchers to draw on, it was decided that the issue take the form of a collection of by-invitation Stakeholder Perspective pieces. As Co-Editor-in-Chief I made the decision to give contributors considerable leeway with respect to format, length and focus because there are no standard formats for this kind of cross-over research in Prosthetics and Orthotics. This was done in order to make it easier for people to contribute to a body of work that is, in most cases, outside of their standard research repertoire or the daily work they do.

What emerged is a collection of thoughtful and varied perspectives in an Edition that is atypical in look, feel and balance. Contributions varying widely in style, length and topic as they were written by a wide spectrum of stakeholders, all of whom provide a unique perspective on the topic in order to create an inclusive picture that respects the diversity of the people effected. The issue is organized around the following stakeholder groups: End-user, Researcher (Engineering and Clinical), Prosthetic and Orthotic Practitioner, Educator and, in a category all on its own, Dr. Frossard's team's work, which was broken down into four papers, in order to present as much of their work as possible in easier to digest portions. I am particularly pleased to include the voices of two stakeholder groups that are very often not given much profile in academic literature in this edition – prosthetic & orthotic clinical practitioners and prosthetic & orthotic device users. Their voices are often excluded despite being profoundly impacted by all decisions made, especially at the funding policy level. The ordering of the papers is deliberate. The issue begins with the highly personal story of an end user who became involved in exoskeleton design when she unexpectedly became disabled. The next section focuses on topics relating to the importance of Data and Business Intelligence. Next are the works for Frossard and his collaborators. Then come a collection of innovative thought and approaches in the sector, followed by a career retrospective from Professor Sir Saeed Zahedi, OBE that *also* looks forward and a final paper which answers Prof Sir Zahedi, and other authors', call to Prosthetic and Orthotic Educators to better prepare graduates for the complex, data driven future that is coming. Interspersed throughout are perspectives from Clinical Practitioners who form the bridge between engineers and innovators and the end user.

The goal of this Special Edition is not to create a snapshot of the current ‘state of the art’ on this topic, but instead to spark discussion where there is an acknowledged knowledge gap, in the hope that it will encourage more researchers to engage in formalized research and publications on this topic. A further atypical feature is that each paper includes a “Call to Action”. Each author was asked to consider what they have written and to identify one or more specific Calls to Action coming from that which they believe would create tangible value and to identify who or what institution has the authority to initiate that action. Sometimes those who see solutions or who ‘feel the pinch’ of a problem are not in a position to enable change. Therefore, it is important, in underserved areas, to both identify potential pathways toward solutions and to highlight who has the ability and authority to make identified changes happen.

## CONCLUSION

I would like to thank all the contributors who took a risk when they responded positively to our invitation to contribute to this eclectic Special Edition. And, I would like to thank the readers of this collection of works who may be challenged by what they read because of the atypical format and content. In laying out the Edition our aim was to challenge readers to think outside the boundaries of their specialty, be it in clinical or engineering, and to spark a discussion on the unique challenges and opportunities that the prosthetic and orthotic market presents. It is only in considering perspectives from the full spectrum of stakeholders that a comprehensive understanding of the Prosthetics and Orthotics, as a whole, can be formed. I sincerely hope that this Special Edition will inspire some readers to delve into this topic in a deeper way, to benefit all stakeholder represented in this edition.

## CALL TO ACTION

I will end this introduction with my own Call to Action, which is that I ask that those persons or institutions identified as having the ability and authority to make change happen seriously deliberate on the calls and the underlying issues that led to their formulation and proceed to act on them.

## DECLARATION OF CONFLICTING INTERESTS

I have no conflicts to interest to declare.

## SOURCES OF SUPPORT

None.
